# Markerless Force Estimation via SuperPoint-SIFT Fusion and Finite Element Analysis: A Sensorless Solution for Deformable Object Manipulation

**DOI:** 10.3390/biomimetics10090600

**Published:** 2025-09-08

**Authors:** Qingqing Xu, Ruoyang Lai, Junqing Yin

**Affiliations:** 1 School of Mechanical and Electrical Engineering, Suqian University, Suqian 223800, China; 21063@squ.edu.cn; 2 Jiangsu Engineering Research Center of Key Technology for Intelligent Manufacturing Equipment, Suqian 223800, China; 3 School of Mechanical and Electrical Engineering, Xi’an Polytechnic University, Xi’an 710600, China; lairuoyang@stu.xpu.edu.cn

**Keywords:** sensorless force detection, force feedback, feature fusion, 3D reconstruction, finite element analysis

## Abstract

Contact-force perception is a critical component of safe robotic grasping. With the rapid advances in embodied intelligence technology, humanoid robots have enhanced their multimodal perception capabilities. Conventional force sensors face limitations, such as complex spatial arrangements, installation challenges at multiple nodes, and potential interference with robotic flexibility. Consequently, these conventional sensors are unsuitable for biomimetic robot requirements in object perception, natural interaction, and agile movement. Therefore, this study proposes a sensorless external force detection method that integrates SuperPoint-Scale Invariant Feature Transform (SIFT) feature extraction with finite element analysis to address force perception challenges. A visual analysis method based on the SuperPoint-SIFT feature fusion algorithm was implemented to reconstruct a three-dimensional displacement field of the target object. Subsequently, the displacement field was mapped to the contact force distribution using finite element modeling. Experimental results demonstrate a mean force estimation error of 7.60% (isotropic) and 8.15% (anisotropic), with RMSE < 8%, validated by flexible pressure sensors. To enhance the model’s reliability, a dual-channel video comparison framework was developed. By analyzing the consistency of the deformation patterns and mechanical responses between the actual compression and finite element simulation video keyframes, the proposed approach provides a novel solution for real-time force perception in robotic interactions. The proposed solution is suitable for applications such as precision assembly and medical robotics, where sensorless force feedback is crucial.

## 1. Introduction

In robotic precision manipulation and industrial automation, secure grasping and precise control critically depend on the synergistic operation of vision-force perception systems. Machine vision systems can effectively acquire the target object state information, including the spatial pose and surface characteristics; however, they cannot capture critical mechanical data during actual grasping procedures. Although conventional pressure-sensor-based force-feedback mechanisms provide reliable contact force information, their hardware dependency restricts their application in specialized scenarios, such as flexible object grasping. To overcome this technical bottleneck, vision-based sensorless force detection methods have recently gained significant attention because they maintain visual state perception while deducing grasping force conditions from visual information [1,2,3,4,5,6]. The visual modality dominates noncontact force estimation, with existing research broadly categorized into deformation-driven and data-driven approaches. While deformation-driven methods establish physical relationships between object deformation and force, they generally suffer from limited applicability: Although Luo and Nelson’s [7] snake-FEM framework improves segmentation rationality and adaptive deformation capability, its reliance on manual contour initialization and sensitivity to image noise hinder automated processing. While Fonkoua et al. [8] enhanced real-time environmental interaction using RGB-D cameras and dynamic FEM, their depth-dependence renders the method ineffective with monocular cameras. Although marker-based methods by Fu et al. and Yu et al. [9] improved external force estimation accuracy, their requirement for artificial markers severely restricts natural interaction scenarios. In contrast, our SuperPoint-SIFT fusion method eliminates dependencies on artificial markers and depth information, enabling feature extraction with standard monocular cameras and significantly improving generalizability.

Data-driven approaches map force information through visual features, yet face several inherent limitations. While pioneering work by Lee [10] has demonstrated the feasibility of human visuo-tactile perception and deep learning has advanced the field, these approaches lack robust quantitative analysis frameworks. Lee et al. [11] developed conditional GANs to generate tactile images from visual inputs, simulating GelSight sensor’s mechanical characterization, albeit requiring extensive annotated datasets for training. Hwang et al. [12] proposed an end-to-end force estimation model based on sequential image deformation, yet its applicability is restricted to scenarios with substantial deformation. Cordeiro et al. [13] innovatively integrated spatiotemporal features of surgical tool trajectories, expanding multimodal visual force estimation boundaries at the cost of significantly increased system complexity. Notably, Zhu et al. [14] revealed that micro-deformations in rigid objects lead to image feature degradation—a fundamental limitation pervasive in vision-only methods. To address this critical challenge, our work innovatively incorporates finite element analysis (FEA) into a visual force estimation framework, leveraging physical modeling to enhance micro-deformation resolution and compensate for data-driven shortcomings. Regarding hybrid methodologies, while Sebastia [15], Liu [16], and Zhang [17] improved force estimation by incorporating proprioceptive information, their reliance on joint torque sensors or motor current measurements prevents genuine sensor-free implementation.

The proposed methodology demonstrates three distinct advantages over existing approaches: (1) Our designed multiscale feature-fusion strategy innovatively combines deep convolutional networks (SuperPoint) with traditional descriptors (SIFT), preserving SIFT’s geometric invariance advantages [18] while harnessing SuperPoint’s semantic perception capabilities [19], thereby substantially improving the feature-matching robustness and computational efficiency in complex environments. (2) We establish a complete closed-loop system, bridging visual features with mechanical analysis. The feature-fusion outputs are transformed into displacement boundary conditions for FEA via 3D displacement field reconstruction, enabling solutions to nonlinear mechanical problems and achieving cross-domain mapping from an image’s feature space to its physical-parameter space. (3) We developed a dynamic video-sequence-based mechanics-detection approach: feature point displacement fields decode object deformation dynamics, and a video similarity comparison mechanism noninvasively validates the spatiotemporal consistency between FEA simulations and real-world deformations. This framework eliminates the reliance on conventional pressure sensors, and requires only standard cameras and algorithms without complex sensors or markers. Its cost-effectiveness and operational simplicity render it particularly suitable for marker-free natural scenarios, offering a universal solution for external force detection in applications such as precision assembly and medical robotics.

The paper is organized as follows: Section 2 first introduces the feature point definition methodology for deformable grasped objects, which serves as the foundation for subsequent feature extraction. Building upon this foundation, we present the SuperPoint-SIFT fused feature extraction algorithm and its corresponding 3D reconstruction method. Subsequently, we detail the proposed vision-based force sensor alternative, comprising (1) a displacement-based FEA contact force-estimation method and (2) a keyframe-feature-guided FEA co-validation approach. Section 3 comprehensively describes the experimental setup and validates the proposed sensorless external force detection method integrating SuperPoint-SIFT feature extraction with FEA. Finally, Section 4 concludes the paper by summarizing its key contributions and outlining potential future improvements.

## 2. Feature Point Definition and Extraction

When reconstructing 3D displacement fields using feature-fusion algorithms, accurate acquisition of the feature information of the target object and grasping parameters is a prerequisite, making the precise definition of object features essential. In general applications, feature points refer to distinctive local structures in images that satisfy certain criteria, such as repeatability, distinctiveness, geometric invariance, and computational efficiency. In the proposed method, critical feature points for deformable object grasping are defined as local image structures that satisfy the following criteria: (1) salient features within the object-gripper contact region or (2) features exhibiting significant spatial or geometric state variations during object deformation. Multiple feature points from the contact region images can be tracked to monitor their displacements across different deformation states or consecutive frames. The displacement vectors of these feature points were then analyzed to quantify the local strain and global deformation patterns on the contact surface using the computed distance relationships within the point set. To validate method generality, we selected standardized black nitrile butadiene rubber (NBR) O-ring gaskets as representative test specimens, using the following specified parameters: outer diameter 65 ± 0.2 mm, cross-section diameter 5 ± 0.1 mm, and Shore A hardness 70 ± 2. These gaskets exhibit smooth surfaces and perfectly circular cross-sections. Although simple, these are representative geometric characteristics that thoroughly validate the capability of the algorithm to extract fundamental geometric features and establish benchmark references for subsequent analyses of complex-shaped objects. Although O-rings were used for validation, the feature fusion algorithm (SuperPoint-SIFT) and FEA framework are inherently generalizable. SuperPoint handles low-texture regions using deep semantic perception, while SIFT ensures geometric invariance, enabling the method to adapt to objects with diverse shapes and materials.

(a) Displacement points of O-ring gaskets.

**Figure 1:**
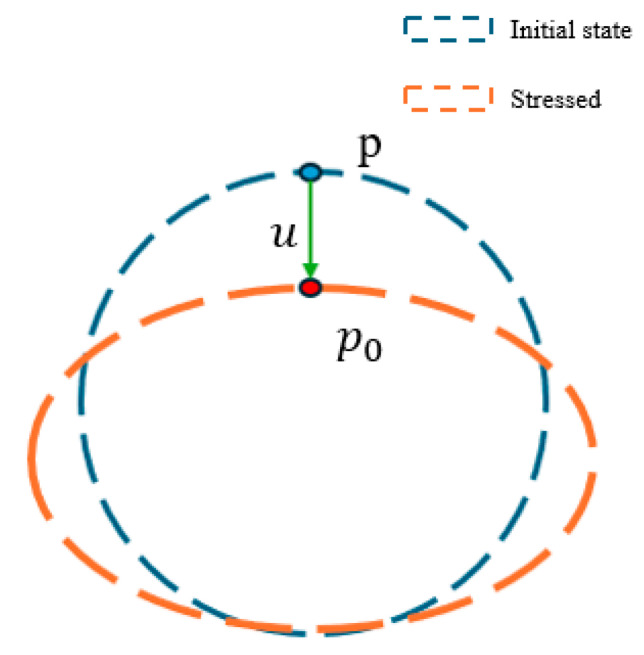
Initial and compression location feature points.

These represent the terminal points of the displacement vectors between the actual positions under external loading and the theoretical positions in the unloaded state. The displacement magnitude can be determined by comparing the measured post-load positions with the initial unloaded positions, as illustrated in Figure 1—that is, displacement point u of the O-ring gaskets characterized the local deformation under loading. We define the feature point displacement vector as the spatial vector difference between the measured deformed position $p_{0}$ and the theoretical undeformed position *P* (both 3D points in the world coordinate system): $u=P-P_{0}$.

(b) Forced bearing contact points.

**Figure 2:**
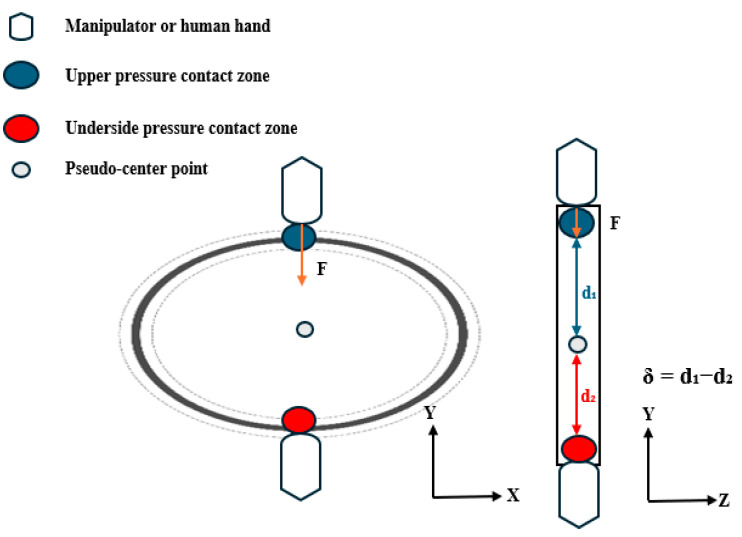
Isotropic contact feature points in the direction of the force.

**Figure 3:**
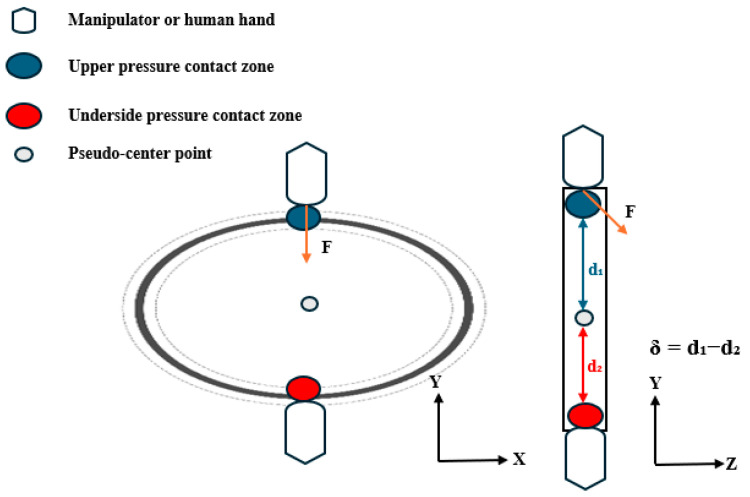
Anisotropic contact feature points in the direction of force.

The contact feature points $C_{u}$ and $C_{d}$ represent the vertex regions formed during the gripper–O-ring interaction, defined as the uppermost and lowermost points of the contact area contour, respectively, as shown in Figure 2 and Figure 3. The pseudo-center-point $C_{pCp}$ is defined as the representative geometric center of the O-ring in our study. The interboundary distance $\left\|C_{u}-C_{d}\right\|$ indicates the contact zone span; the reduction in this zone under a constant contact area shows a positive correlation with the applied pressure.

Pressure distribution symmetry serves as a critical indicator for evaluating the vertical equilibrium. We quantify the symmetry by computing the offset *δ* between upper/lower pressure centroids ($C_{u}/C_{d}$) and the pseudo-center as follows:

(1) $$\delta =\left\|C_{u}-C_{pCp}\right\|-\left\|C_{d}-C_{pCp}\right\|$$

$C_{u}$ and $C_{d}$ denote the coordinate positions of the upper and lower contact pressure center points, respectively, while $C_{pCp}$ represents the system-defined pseudo-center reference point.

Under compression, the O-rings develop localized indentation deformations (forming contact feature points), and the spatial distributions of these deformations provide crucial grasp state indicators. Pressure distributions exhibit two modes: isotropic (symmetric centroids creating uniform stress fields) and anisotropic (asymmetric deformation from the centroid offset causing non-uniform stresses). The gasket’s mechanical state is quantified using both 3D feature-point distributions and *δ*: when *δ* ≈ 0, coincident centroids indicate equilibrium with axisymmetric indentation and uniform annular contact, and increasing *δ* values reflect growing asymmetry; these mechanical characteristics enable a discriminative grasp-state assessment.

When robotic/human hands grasp objects, HSV color-space segmentation is employed due to its perceptual uniformity and robustness to illumination variations. Unlike RGB, HSV separates luminance (*V*) from chrominance (*H* and *S*), which minimizes the impact of lighting changes during the grasping process. This method provides reliable foreground masking for feature extraction in the environment.

(2) $$M(x,y)=\left\{\begin{array}{ll} 1 & \mathrm{if}\;H_{\min}\leq H(x,y)\leq H_{\max}\;\mathrm{and}\;S_{\min}\leq S(x,y)\leq S_{\max}\;\mathrm{and}\;V_{\min}\leq V(x,y)\leq V_{\max}\\ 0 & \mathrm{otherwise} \end{array}\right.$$

The functionM(*x*,*y*) outputs 1 when a pixel’s hue H(*x*,*y*), saturation S(*x*,*y*), and brightness V(*x*,*y*) simultaneously fall within their respective threshold ranges, [$H_{max}$, $H_{min}$], [$S_{max}$, $S_{min}$], and [$V_{max}$, $V_{min}$], and 0 otherwise.

### 2.1. Fusion Feature Extraction and 3D Reconstruction

**Figure 4:**
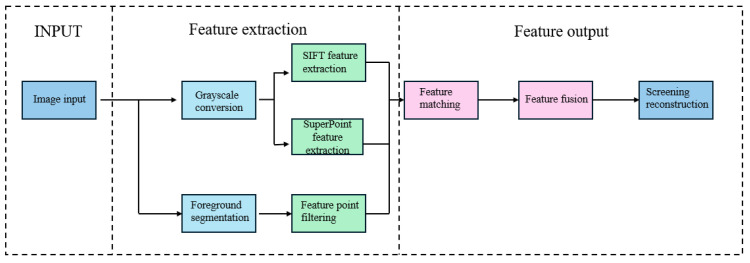
Flowchart of feature fusion extraction.

Figure 4 illustrates the feature fusion extraction and displacement field reconstruction pipeline, where the output displacement vector is considered as the boundary condition for mechanical estimation. The integrated feature extraction and 3D reconstruction approach comprises four key steps [18]: (1) SuperPoint-SIFT feature detection, (2) feature point matching, (3) essential/fundamental matrix estimation via epipolar geometry, and (4) triangulation-based 3D point-cloud reconstruction. Our approach synergizes traditional SIFT features with deep learning-based SuperPoint features to enhance feature extraction for subsequent 3D reconstruction.

To address the feature-matching challenges in markerless low-texture object deformation detection, we independently extracted SuperPoint features (for low-texture regions) and SIFT features (for texture-rich regions) within segmented ROIs and subsequently merged them using a late-fusion strategy. The pipeline involves (1) independent feature points and descriptor extraction using SuperPoint [18] and SIFT, (2) spatial filtering via shared foreground masks, (3) k-nearest neighbor matching with Lowe’s ratio test for both feature types, and (4) merged match-pair refinement using RANSAC [19].

**Figure 5:**
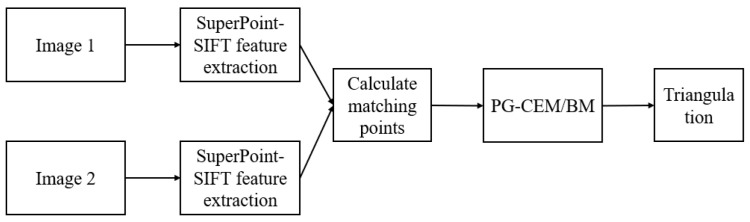
Flow chart of 3D reconstruction.

Figure 5 illustrates the two-view stereo vision pipeline used for 3D reconstruction [20]: using matched feature pairs and camera calibration parameters, we implemented structure-from-motion (SfM) [21] via OpenCV’s findEssentialMat and recoverPose functions, followed by dense point cloud reconstruction using triangulatePoints [22]. This process adheres to standard epipolar constraints and triangulation principles [23], thereby minimizing reprojection errors for pose estimation and 3D point optimization.

### 2.2. Contact Force Estimation Based on Displacement and Finite Element Analysis

**Figure 6:**
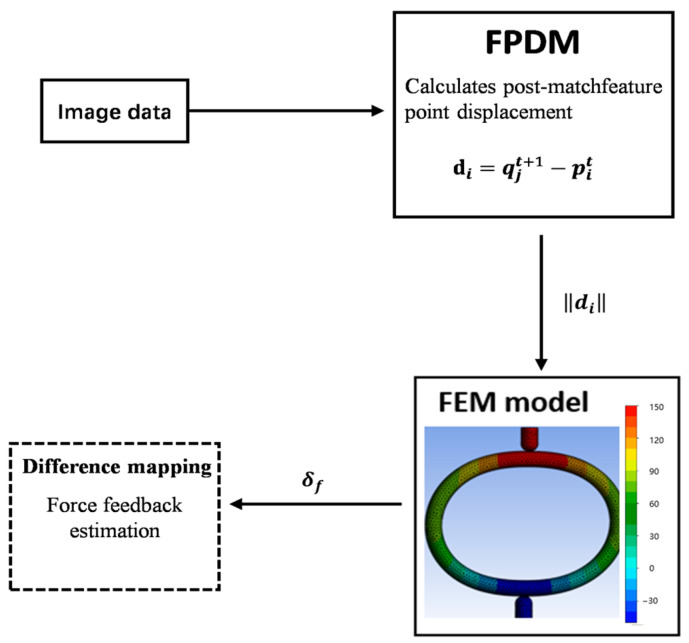
Estimation of displacement and finite element contact force.

The proposed method acquires the displacement variations in the surface feature points through 3D reconstruction, which are then input as boundary conditions into a finite element model. Integration with feature-fusion algorithms enables contact-force computations, as shown in Figure 6.

The Feature Point Displacement Module (FPDM) computes the displacements of the matched feature points, taking pre- and post-deformation 3D point clouds as inputs, and outputs the mean displacement vector $\left\|d_{i}\right\|$ of the matched point pairs as the FEA boundary conditions. Here, $\left\|d_{i}\right\|$ denotes the feature point displacement vector and $\delta_{f}$ represents the stress computed by the FEM based on these displacement vectors.

Following feature fusion and 3D reconstruction, the deformation displacement was quantified by establishing precise correspondence between the pre- and post-deformation point clouds. For matched point pairs ($p_{i}^{t},\;q_{j}^{t+1})$, where $p_{i}^{t}$ denotes the i-th point in the pre-deformation cloud $P_{t}$ and $q_{j}^{t+1}$ denotes the j-th matched point in the post-deformation cloud $P^{t+1}$, the displacement vector $d_{i}$ is computed as follows:

(3) $$d_{i}=q_{j}^{t+1}-p_{i}^{t}=\left[\begin{array}{c} q_{j}^{t+1,x}-p_{i}^{t,x}\\ q_{j}^{t+1,y}-p_{i}^{t,y}\\ q_{j}^{t+1,z}-p_{i}^{t,z} \end{array}\right]$$

This vector explicitly captures both the direction and magnitude of the 3D displacement. To quantify the displacement amplitude, we computed the Euclidean norm (geometric length) as follows:

(4) $$\left\|d_{i}\right\|=\sqrt{{\left(q_{j}^{t+1,x}-p_{i}^{t,x}\right)}^{2}+{\left(q_{j}^{t+1,y}-p_{i}^{t,y}\right)}^{2}+{\left(q_{j}^{t+1,z}-p_{i}^{t,z}\right)}^{2}}$$

Superscripts *x*, *y*, and *z* denote the three coordinate components. This computation captured both local displacements and global deformation patterns. The obtained surface displacement vectors $d_{i}\;$ serve as boundary conditions for the finite element model. We employed a hyperelastic constitutive model with the Mooney–Rivlin strain energy function to characterize the rubber material properties as follows:

(5) $$W=C_{10}\left({\bar{I}}_{1}-3\right)+C_{01}\left({\bar{I}}_{2}-3\right)+\frac{1}{D}(J-1{)}^{2}$$

The Mooney–Rivlin model was selected for its capability to accurately characterize the deformation behavior of rubber-like materials, where W is the strain energy density, $C_{10}$ and $C_{01}$ are material constants; ${\bar{I}}_{1}$ and ${\bar{I}}_{2}$ denote the first and second deviatoric strain invariants, representing the isochoric deformation resistance and shape-change resistance, respectively, and *J* indicates the volume ratio (*J* < 1 corresponds to compression). These parameters were calibrated using uniaxial compression tests on NBR O-rings to ensure physical accuracy. Finite element discretization yields a nonlinear equation, K(u)u=F, where K is the stiffness matrix and F is the nodal force vector. The Newton–Raphson iteration [8] solves these equations to obtain the displacement-stress responses. A precomputed FEM forward simulation database establishes a mapping between the feature point displacements and contact forces, enabling direct force estimation via displacement-based interpolation.

The proposed framework establishes a nonlinear displacement-stress field mapping, creating a complete vision–mechanics coupling system that enables FEM-based force detection solely through feature point deformation observations.

### 2.3. Based on the Keyframe Feature Point-Finite Element Collaborative Comparison Method

The video temporal alignment begins with uniform frame sampling $\Delta =\left\lfloor \frac{N}{k}\right\rfloor$, where the i-th keyframe position $F_{i}=1+\left(i-1\right)\times \Delta ,i=1,2,\ldots ,k$, which eliminates segmentation-dependent noise by extracting ten keyframes without relying on shot-boundary detection. This enables localized feature similarity computation between video sequences. The extracted frames underwent a three-stage similarity validation: (1) feature matching using the Euclidean distance, (2) point cloud registration via Iterative Closest Point (ICP), and (3) similarity assessment. This pipeline robustly bridges the 2D image features for 3D geometric verification.

During feature matching, given two feature sets$\;F_{1}={\left\{f_{i}^{1}\right\}}_{i=1}^{n}$ and${\;F}_{2}={\left\{f_{j}^{2}\right\}}_{j=1}^{m}$, we established a cross-view correspondence by performing a k-nearest neighbor search (*k* = 2) [24] for each query feature in the target descriptor space.

(6) $$\mathrm{NN}\left(f_{i}^{*}\right)=\left\{\left(f_{h}^{2},d_{1}\right),\left(f_{j_{2}}^{2},d_{2}\right)\right\}$$

**Figure 7:**
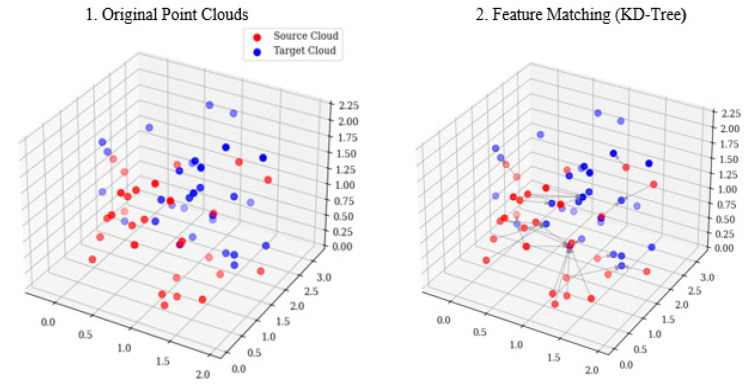
KD-tree nearest-neighbor matching process.

Here, *d*_1_ and *d*_2_ denote the distances to the first- and second-nearest neighbors, respectively. The matches output contains correspondence lists with DMatch objects indicating best/second matches in the target images, where smaller distances imply higher feature similarity. Point cloud registration aligns 3D features from multiple views into a unified coordinate system via (1) a KD-tree-based nearest-neighbor search to establish the initial correspondence between point clouds *P*_1_ and *P*_2_, where each *p* ∈ *P*_1_ finds its closest point in *P*_2_, as shown in Figure 7, and (2) computing optimal rigid transformation (rotation *R* and translation *t*) via SVD by minimizing the squared Euclidean distance, $\min_{R,t}\sum_{i=1}^{N}{\left\|R\cdot p_{i}+t-q_{i}\right\|}^{2}$, where *p*_1_*_i_* ∈ *P*_1_ and *p*_2_*_i_* ∈ *P*_2_ are matched pairs. The derived transform optimally aligns the point clouds [25].

The similarity assessment employs the mean Euclidean distance between registered point clouds as follows:

(7) $$\bar{d}=\frac{1}{N}\sum_{i=1}^{N}\;{\left\|T\left(p_{i}\right)-q_{i}\right\|}_{2}$$

where *T* denotes the estimated rigid transformation, $p_{i}$, and $q_{i}$ are the matched point pairs, and *N* is the number of valid correspondences. To mitigate scale variation, we defined the normalized similarity score as follows:

(8) $$\begin{array}{c} s=1-\frac{\bar{d}}{d_{max}}\; \end{array}$$

Here, $d_{max}=\underset{p,q}{max}\;\|p-q{\|}_{2}$ represents maximum inter-cloud distance, normalizing similarity to [0, 1] (1: perfect alignment; 0: no match).

High similarity scores indicate geometric deformation consistency between the real frames and FEA simulations, thereby providing foundational evidence for establishing cross-modal mapping. When the geometric similarity exceeds the thresholds, real-world deformations spatially correspond to the FEA predictions, enabling accurate stress/displacement field transfer from simulations to physical states.

**Figure 8:**
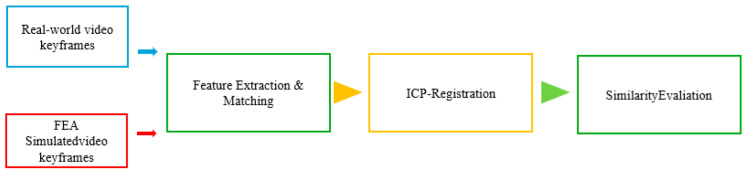
Comparison of finite element collaboration based on keyframe feature points.

The proposed method concurrently acquires experimental videos of the compressed objects and their corresponding FEA simulations, achieving spatiotemporal alignment through uniform temporal sampling and spatial point cloud registration. Video similarity matching establishes real-to-simulation mappings, ultimately enabling vision-based state estimation without a physical sensor, as shown in Figure 8.

## 3. Experimental Verification

Based on the material constitutive relationships, we adopted the Mooney–Rivlin hyperelastic model to characterize the mechanical behavior of the rubber. According to Mooney’s seminal work [15], nonlinear finite element problems can be categorized into three types: (1) material nonlinearity, (2) large displacement/rotation with a small strain, and (3) large displacement/rotation with a large strain.

### 3.1. Deformation and Force Feedback Experiments

**Figure 9:**
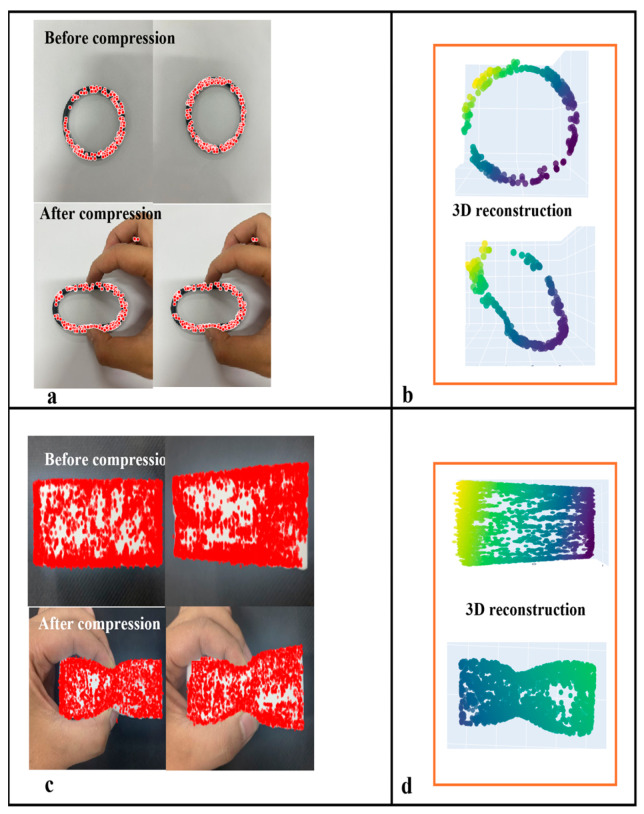
(**a**) Characteristic points of O-ring before and after compression; (**b**) 3D reconstruction of O-ring characteristic points before and after compression; (**c**) Characteristic points of sponge block before and after compression; (**d**) 3D reconstruction of sponge block characteristic points before and after compression.Three-dimensional reconstruction from two perspectives.

Displacement–stress mapping was established via finite element simulations under displacement-controlled compression. That is, a fixed constraint was applied at the bottom, with manually applied displacement loads (0–30 mm isotropic/0–60 mm anisotropic) in 20 loading steps. Hex-dominant meshing with local refinement (size: 0.5 mm) at contact zones was employed to precisely capture the contact stress distributions. The augmented Lagrangian method ensured computational convergence. SuperPoint-SIFT extracts features from the pre-and post-compression images. Three-dimensional displacements were computed via binocular reconstruction (6° viewing angle, approximating human parallax) [26] and input to FEM for stress $\delta_{f}$ calculation at loading positions. The experimental data show that a 13.78 mm feature displacement in the O-ring specimens corresponds to a 5.9 N contact force via FEM conversion, as shown in Figure 9a.

**Figure 10:**
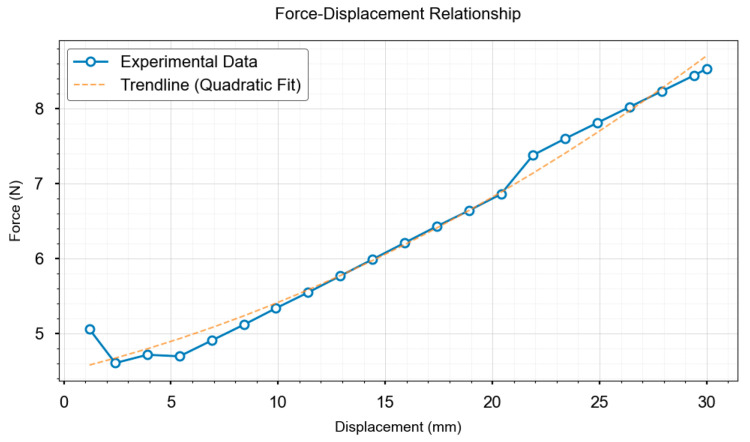
Displacement–stress data of a rubber gasket with an isotropic force direction.

**Figure 11:**
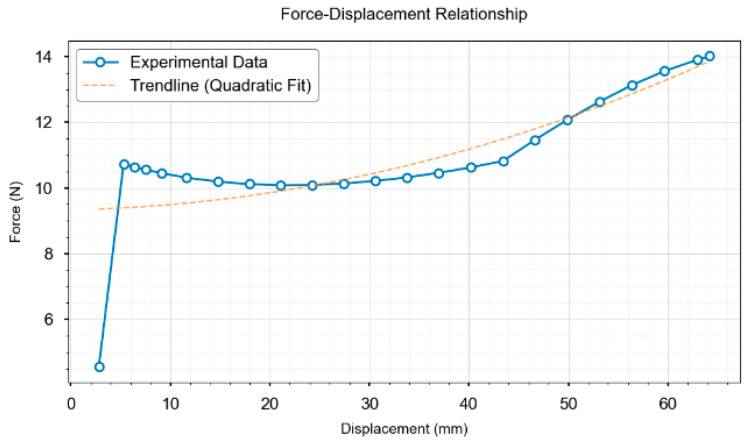
Displacement–stress data of a rubber gasket with anisotropic force direction.

Displacement–force data pairs were plotted to establish quantitative correlations, as shown in Figure 10 and Figure 11. The extracted $\left\|d_{i}\right\|$ parameters were interpolated using pre-calibrated force–parameter curves for local stress prediction. These curves, obtained from FEM simulations, map $\left\|d_{i}\right\|$ to actual stress$\;\delta_{f}$ under specified conditions.

**Figure 12:**
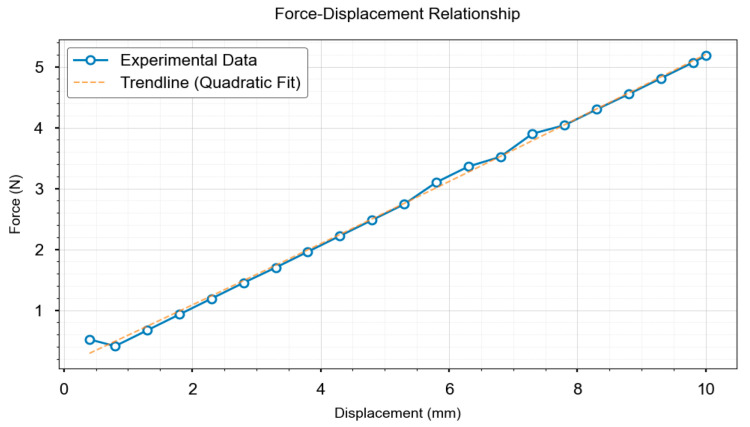
Displacement–stress data for sponge blocks.

To demonstrate the generalizability of the proposed method, experimental validation was conducted using a sponge block. A displacement-stress mapping relationship was similarly established using finite element simulation. The compression process was simulated using displacement control, consistent with the aforementioned experimental procedure. The contact region employed a hexahedron-dominant mesh with local refinement (element size: 0.5 mm) to accurately capture contact stress distribution. Experimental data indicate a 6.2 mm displacement at the characteristic point of the sponge block, which corresponds to a force value of 3.25 N derived from finite element analysis, as shown in Figure 9c. The displacement-stress data pairs for the sponge block are plotted as a line chart, shown in Figure 12.

The experimental results demonstrate that our vision-based deformation force estimation method fundamentally operates by inferring contact forces through the observation of geometric variations at the contact interface. This principle remains applicable to sliding or rotational scenarios, provided that robust visual tracking algorithms capable of handling motion blur and occlusion challenges are implemented.

**Figure 13:**
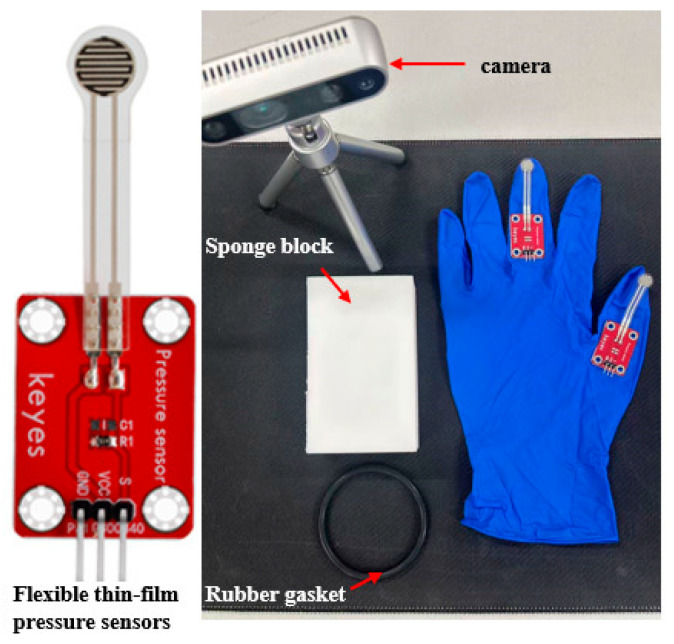
Flexible-pressure sensor test equipment.

**Figure 14:**
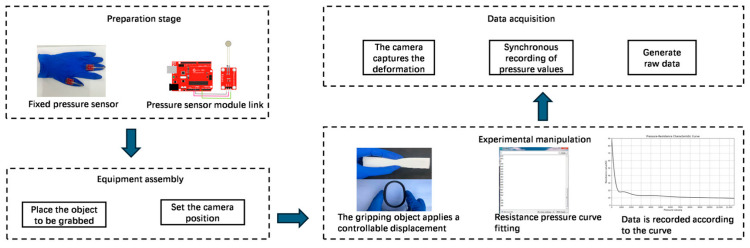
Test process for flexible-pressure sensors.

The validation employed flexible thin-film pressure sensor gloves for experimental contact pressure measurements [27]. Keyes pressure sensors (0–5 kg range) exhibited a repeatability error of ±9.7%, consistency deviation of ±10%, sensitivity of 150 g, and response time of <1 ms. Sensor calibration uses voltage-divider circuits (510 kΩ resistor) with an analog signal *A*0, computing real-time resistance via *R* = (1023 − *A*0) × 510/*A*0. As illustrated in Figure 13 and Figure 14, the O-ring specimen was horizontally compressed between the sensor-gloved indenter and the platform until the predetermined displacement was achieved. Real-time resistance monitoring enabled the acquisition of actual contact forces on the O-ring, with the same methodology applied to validate sponge block specimens.

The flex-sensor force represents the physically measured contact force, while the FE-inverse force denotes the O-ring and sponge block reaction forces computed via finite element analysis. Experimental results demonstrate that our vision-based deformation force estimation method fundamentally infers contact forces by analyzing geometric variations at contact interfaces. This principle remains applicable to sliding or rotational scenarios, provided that robust visual tracking algorithms are employed to address motion blur and occlusion challenges. As demonstrated in Table 1, Table 2 and Table 3, the experimental results confirm that the proposed method achieves high predictive accuracy and robustness. Under isotropic compression (30 mm displacement), the O-ring test yielded a predicted force of 8.53 N versus a measured value of 9.21 N, corresponding to a 7.38% error. Across the 10–30 mm displacement range, the mean error was 7.60% with an RMSE of 7.62%, peaking at 8.32% at 25 mm displacement. For anisotropic compression (60 mm displacement), the mean error was 8.15% with an RMSE of 7.37%, reaching a peak error of 9.96% at 40 mm displacement. In sponge block compression tests (10 mm displacement), the mean error was 7.88% with an RMSE of 7.89%, exhibiting the peak error at the maximum 10 mm displacement. Error analysis revealed the following contributing factors: (1) inherent sensor error (±9.7%), (2) ±5 mm displacement matching error causing ±7.54% force deviation, (3) error propagation from camera calibration and feature matching, (4) finite element modeling inaccuracies, and (5) non-parallel contact surfaces (peak error contribution of 9.96% in anisotropic tests). These systematic errors collectively contributed to prediction deviations.

### 3.2. Based on the Collaborative Comparison of Keyframe Feature Points and Finite Elements

**Figure 15:**
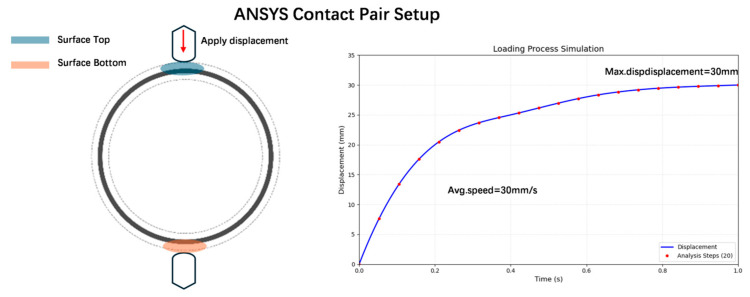
Finite element setting.

O-ring gaskets exhibit a uniform elastic modulus and predictable mechanical response, enabling the accurate simulation of contact pressure distribution; consequently, they were selected as test specimens for subsequent experimental analysis. Manual pressure was applied exclusively to the top of the O-ring, whereas the bottom region remained load-free. In ANSYS(2023R1), as shown in Figure 15, the O-ring surfaces were partitioned into discrete contact pairs—that is, the top and bottom surfaces. Elliptical pressure distribution (*d* ≈ 5 mm) models typical manual contact, with fixed constraints on the surface–bottom, simulating a rigid support. Stress animations were exported for analysis. Maximum displacements of 30 and 60 mm were implemented in 20 loading steps (1 s duration), achieving average compression rates of 30 mm/s and 60 mm/s, respectively, which are consistent with the experimental measurements.

**Figure 16:**
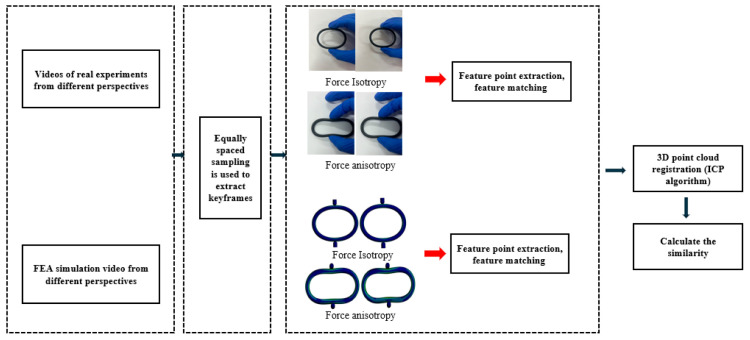
Collaborative comparison experiment of the keyframe feature points and the finite elements.

For experimental FEM video comparisons, ten keyframes were uniformly sampled from both view recordings to assess the feature-match similarity, as shown in Figure 16 Frame sampling followed N/10 intervals (where N is the total number of frames) [28] to ensure temporal uniformity throughout the compression process. SuperPoint-SIFT features were extracted for each keyframe. The Euclidean distance and ICP algorithms enabled 3D reconstruction through (1) feature matching, (2) registration, and (3) similarity assessment, establishing experimental–simulation correspondences via descriptor similarity.

**Figure 17:**
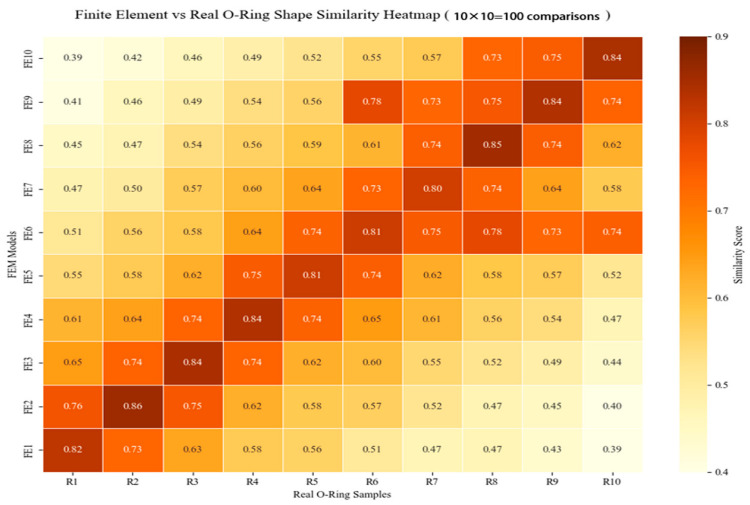
Heat map of force direction isotropy versus similarity.

**Figure 18:**
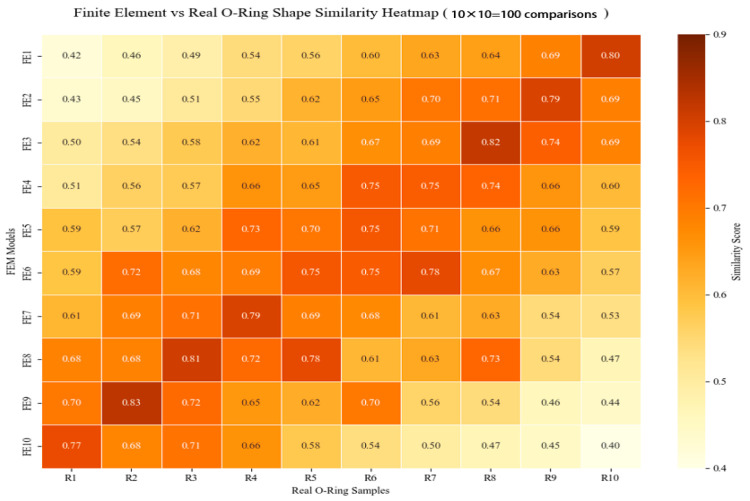
Heat map of force direction anisotropy versus similarity.

In Figure 17 and Figure 18, FE1–10 denote the FEM simulation frames and R1–10 represent the experimental frames. Similarity scores combine the Euclidean feature distance and ICP registration error. For isotropic loading, feature matching achieved 86% similarity between the experimental Frame R2 and FEM Frame FE2. The anisotropic cases showed 83% similarity (R4 and FE4), confirming the deformation pattern correspondence. A high similarity indicates congruence between the actual O-ring deformation in the physical recordings and the simulated stress–strain fields and displacement distributions in the FEM frames. Specifically, the FEM Frame FE4 provides valid references for the stress concentration zones, surface strain patterns, and displacement vector fields observed in synchronized experimental frames. This correspondence (1) validates the FEM’s accuracy under the given conditions and (2) establishes a temporal alignment for simulation-to-experiment mapping, confirming the synchronous mechanical responses in both systems.

Certain discrepancies were observed in the experimental data, as illustrated in Figure 17 (R6-FE9) and Figure 18 (R7-FE5). Our analysis identified two primary sources of error: First, imperfect synchronization occurred during the manual alignment process between the simulated and experimental videos, particularly in their initial timestamps and sampling rates. Second, misalignment of critical feature points (e.g., maximum displacement points) introduced deviations in the similarity computation results.

## 4. Conclusions

Conventional pressure sensors face limitations around hardware deployment and flexible scenario adaptation, whereas current vision-based force-estimation methods still exhibit insufficient robustness under markerless, low-texture, and microdeformation conditions. This study proposes a sensorless force-detection framework that integrates SuperPoint-SIFT feature fusion with FEA, enabling external force estimation by using 3D reconstruction and video feature comparison.

By combining SuperPoint’s semantic awareness and SIFT’s geometric invariance, we enhanced feature-matching robustness in markerless and low-texture scenarios. The novel vision-displacement FEA-fusion framework achieved sensorless contact force estimation with a mean error of <10 %. A video-based deformation dynamics analysis and similarity comparison validated the spatiotemporal consistency between the FEA predictions and actual deformations, establishing a reliable sensorless verification framework. The method is effective under markerless monocular conditions, estimating external stress distributions solely using visual data with notable generalizability. Although offline FEA reduces computational burden, the recalibration of material parameter changes (e.g., nonlinear hyperelasticity) substantially limits online adaptability. Current limitations include (1) robustness under extreme illumination/occlusion, (2) generalizability to complex geometries or heterogeneous materials, and (3) real-time performance for highly dynamic interactions. Future work will focus on (1) developing online material identification and ML-based FEA surrogate models to reduce parameter dependency and (2) optimizing algorithms with parallel computing for real-time performance. (3) In human–robot interaction applications, this method serves as a cost-effective alternative to conventional torque sensors, being particularly suitable for cost-sensitive and non-extreme high-speed collaborative scenarios such as delicate object manipulation and low-speed assembly tasks. However, under the current framework, its response latency and computational delays in highly dynamic operations (e.g., rapid grasping or sudden collision response) require further improvements through algorithmic acceleration and hardware co-optimization to fully meet the demands of high-accuracy real-time human–robot interactions. Despite these limitations, our method innovatively provides sensorless force perception, showing significant value in precision assembly and surgical robotics, which require high-accuracy force control. Further optimization can overcome the limitations of traditional force-sensing and advanced robotic dexterous manipulation technologies.

## Figures and Tables

**Table 1:** Comparison of the actual value of force direction isotropy with the finite element value.

Displacement Value (mm)	Flex-Sensor Force (N)	FE-Inverse Force (N)	Relative Error (%)
10	5.78	5.35	7.44
15	6.57	6.08	7.46
20	7.34	6.80	7.36
25	8.53	7.82	8.32
30	9.21	8.53	7.38

**Table 2:** Comparison of the actual value of the force direction anisotropy with the finite element value.

Displacement Value (mm)	Flex-Sensor Force(N)	FE-Inverse Force (N)	Relative Error (%)
10	10.73	10.12	6.03
20	9.52	10.34	7.93
30	11.25	10.42	7.97
40	12.36	11.24	9.96
50	11.46	12.58	8.90

**Table 3:** Comparison of the actual value of the sponge block with the finite element value.

Displacement Value (mm)	Flex-Sensor Force(N)	FE-Inverse Force (N)	Relative Error (%)
5	2.39	2.58	7.61
8	3.84	4.14	7.82
10	4.79	5.18	8.21

## Data Availability

The original contributions presented in this study are included in the article. Further inquiries can be directed to the corresponding author.
